# Supplementary material for: Structural and functional analysis of LIM domain-dependent recruitment of paxillin to αvβ3 integrin-positive focal adhesions
Source: Commun Biol. 2021 Mar 29;4:380. doi: 10.1038/s42003-021-01886-9 (PMC8007706; doi:10.1038/s42003-021-01886-9)
Supplement: Supplementary file 2 — Supplementary Information [file 42003_2021_1886_MOESM2_ESM.pdf]

Structural and functional analysis of LIM domain-dependent recruitment of paxillin to αvβ3 integrin-positive focal adhesions

Marta Ripamonti[1], Nicolas Liaudet[2], Latifeh Azizi[3], Daniel Bouvard[4], Vesa P. Hytönen[3,5] and Bernhard Wehrle-Haller[1*]

[1]Department of Cell Physiology and Metabolism, University of Geneva, Centre Médical Universitaire, Rue Michel-Servet 1, 1211 Geneva 4, Switzerland.

[2] Bioimaging Core Facility, Faculty of Medicine, University of Geneva, 1211 Geneva 4, Switzerland.

[3]Faculty of Medicine and Health Technology, Tampere University, Kauppi Campus, Arvo Ylpön katu 34, 33520 Tampere, Finland.

[4]Montpellier Cell Biology Research Center (CRBM), University of Montpellier, CNRS, 1919 Route de Mende, 34293 Montpellier, France.

[5]Fimlab Laboratories, Tampere, Finland.

*Corresponding author,
Bernhard.Wehrle-Haller@unige.ch.

Supplementary Figures

Supplementary Fig. 1: Full, uncropped blot images of panels presented in main Figures.

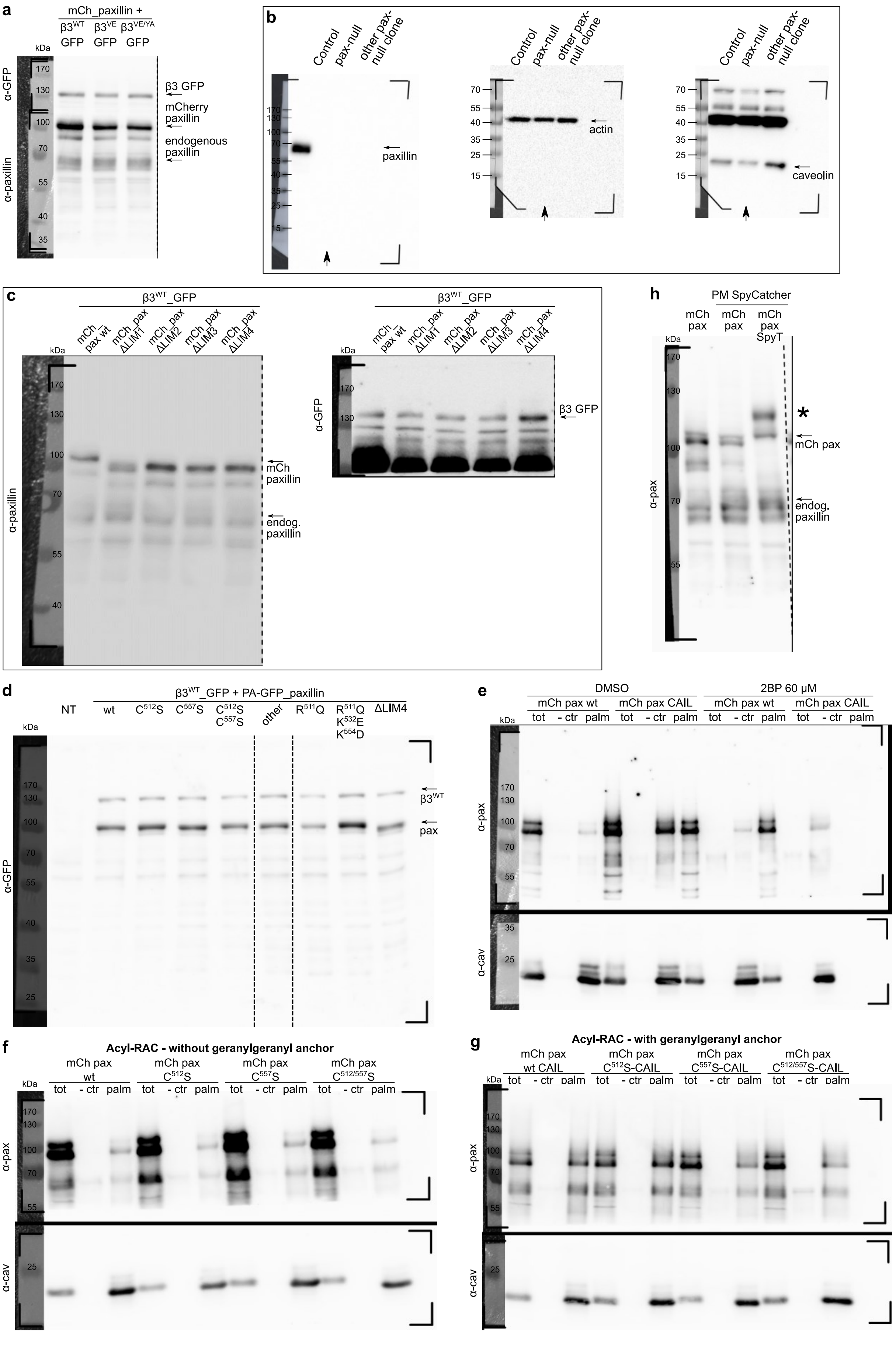

(**a**) NIH-3T3 cells transiently transfected with β3_GFP integrins, wild-type or mutants, and mCherry_paxillin. Recombinant protein expression assessed by western blotting against GFP and total paxillin. Membrane was cut between the 100 kDa and 130 kDa marker bands prior to incubation with antibodies. (**b**) WB of the paxillin-null clone used in this study (arrow) and the parental cells. The membrane was probed with the antibodies indicated without stripping, hence the appearance of previously-detected signals. (**c**) Western blot of protein extracts from NIH-3T3 fibroblasts transiently co-expressing $\beta3^{WT}$_GFP and mCherry-tagged paxillin proteins, wt or deletion mutants. Membrane was firstly probed for paxillin detection, then cut at the level of the 100 kDa marker band and incubated the α-GFP antibody. The paxillin signal, although cropped, was still detected at the bottom of the membrane (no stripping was performed and both primary antibodies were murine). (**d**) Expression analysis of paxillin LIM4 mutants *via* western blot. NIH-3T3 cells were co-transfected with $\beta3^{WT}$_GFP and PA-GFP-tagged paxillin proteins, afterwards both detected by means of the same α-GFP antibody. (**e**) Acyl-Resin Assisted Capture assays, from cell transiently transfected with mCherry_paxillin wt or mCherry_paxillin-CAIL, untreated (DMSO) or treated with 2-bromopalmitate. Uncropped blot corresponding to Fig.8e. Membrane was cut below the 55 kDa and above the 35 kDa marker bands prior to incubation with antibodies. (**f**) Acyl-Resin Assisted Capture assays, from cell transiently transfected with mCherry_paxillin wt or Cys-mutants (without the geranylgeranyl-specifying motif). Uncropped blot corresponding to Fig.8f top. Membrane was cut below the 55 kDa and above the 25 kDa marker bands prior to incubation with antibodies. (**g**) Acyl-Resin Assisted Capture assays, from cell transiently transfected with mCherry_paxillin wt or Cys-mutants geranylgeranylated. Uncropped blot corresponding to Fig.8f bottom. Membrane was cut below the 55 kDa and above the 25 kDa marker bands prior to incubation with antibodies. (**h**) Western blot showing the efficiency of the SpyTag/SpyCatcher technology. * indicates the fusion protein originated by the isopeptide bond formation between the mCherry_paxillin_SpyTag and the PM_SpyCatcher. Uncropped blot corresponding to Fig.9g, therefore showing also the reactivity of the α-paxillin antibody against the endogenous protein. Dotted lines exclude samples not related to this manuscript.

Supplementary Fig. 2: Paxillin, β3 and β1 integrins localization.

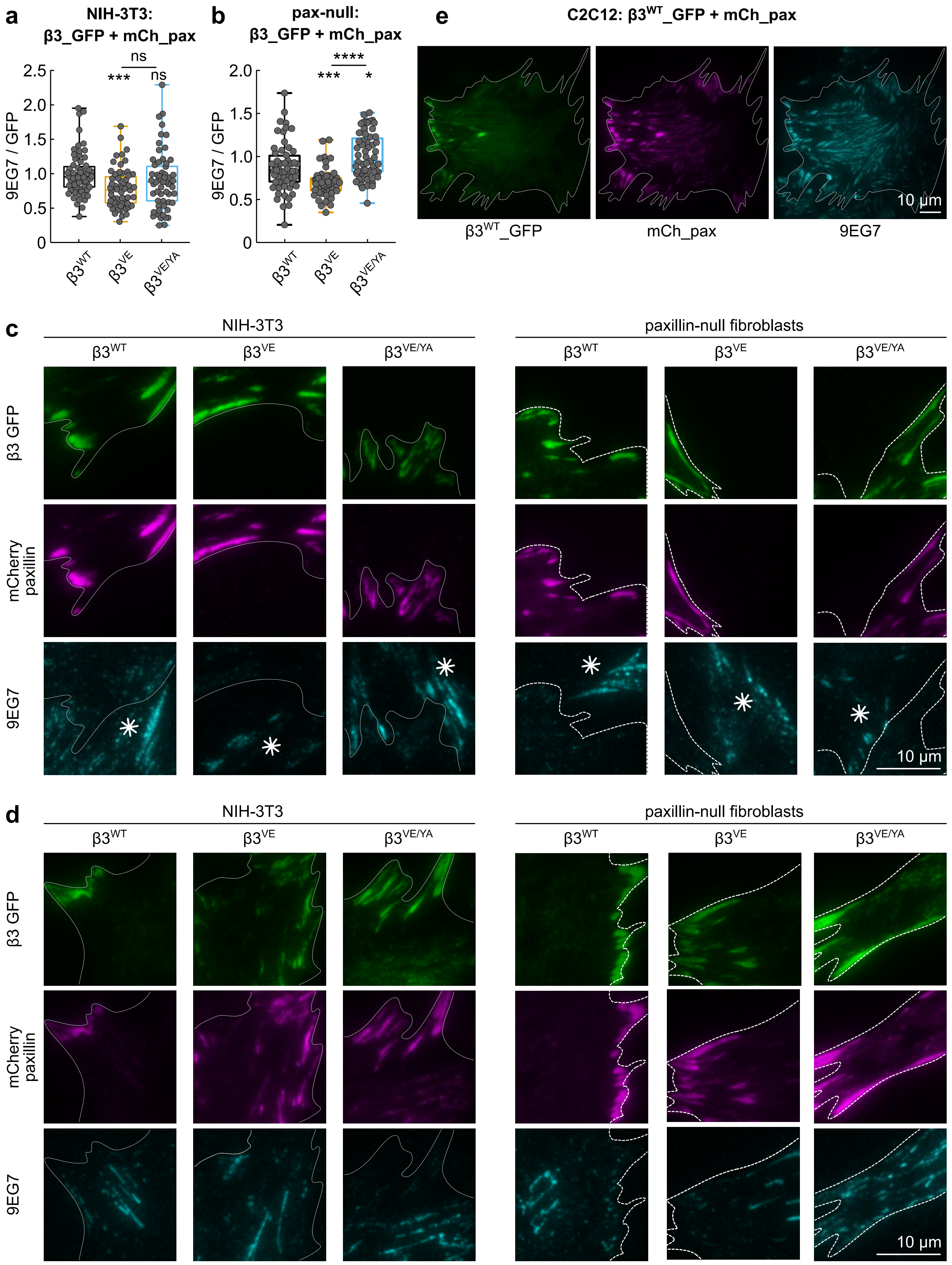

(**a**,**b**) Quantification of the 9EG7 staining within GFP-positive adhesions, expressed as 9EG7/GFP fluorescence ratio in (**a**) NIH-3T3 and (**b**) paxillin-null fibroblasts, co-expressing mCherry_paxillin. The fluorescence ratio was set as 1 in the wild-type condition. Statistical analysis is provided in

Supplementary Dataset 1. (**c**) Representative TIRF images of the 9EG7 staining of NIH-3T3 (left) and paxillin-null (right) fibroblasts, showing the different activation of β1 integrins between neighboring transfected and non-transfected cells (identified by asterisks). (**d**) Representative TIRF images of the 9EG7 staining of NIH-3T3 (left) and paxillin-null (right) fibroblasts, showing the differential localization of the activated β1 integrin receptors between peripheral FAs and central fibrillar adhesions. (**e**) Representative TIRF image of a C2C12 myofibroblast co-expressing $\beta3^{WT}$_GFP and mCherry_paxillin, stained with the 9EG7 mAb.

ns (not significant) $p > 0.05$; *, $p \leq 0.05$; **, $p < 0.01$; ***, $p < 0.001$; ****, $p < 0.0001$. Sample size, complete statistical analysis and p values are provided in tables in supplementary datasets.

Supplementary Fig. 3: β3 integrin variants and adhesion morphology.

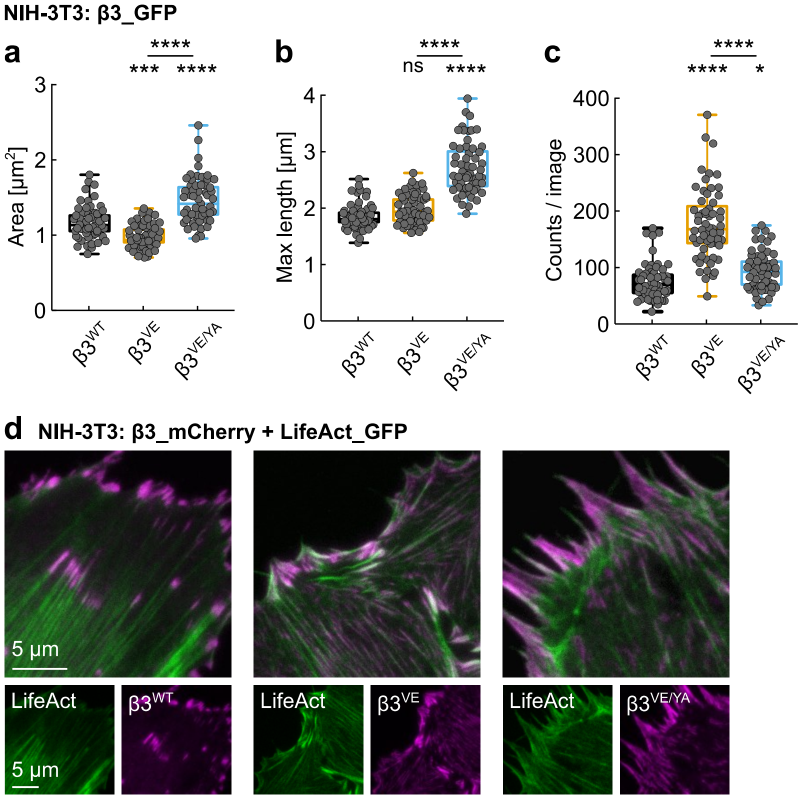

(**a**,**b**) Quantification of the (**a**) area and (**b**) maximal length of β3 integrin-positive adhesions in NIH-3T3 cells expressing the β3_GFP integrin variants, expressed as mean value per acquisition. Statistical analysis is provided in Supplementary Dataset 1. (**c**) Number of β3 integrin-positive adhesions per acquisition in NIH-3T3 expressing the β3_GFP integrin variants, expressed as mean value per acquisition. Statistical analysis is provided in Supplementary Dataset 1. (**d**) Representative TIRF images of NIH-3T3 cells co-expressing GFP-tagged LifeAct and the mCherry-labeled β3 integrin variants used in this study.

ns (not significant) $p > 0.05$; *, $p \leq 0.05$; **, $p < 0.01$; ***, $p < 0.001$; ****, $p < 0.0001$. Sample size, complete statistical analysis and p values are provided in tables in supplementary datasets.

Supplementary Fig. 4: BiFC and SpyTag/SpyCatcher assays to study focal adhesions organization and function.

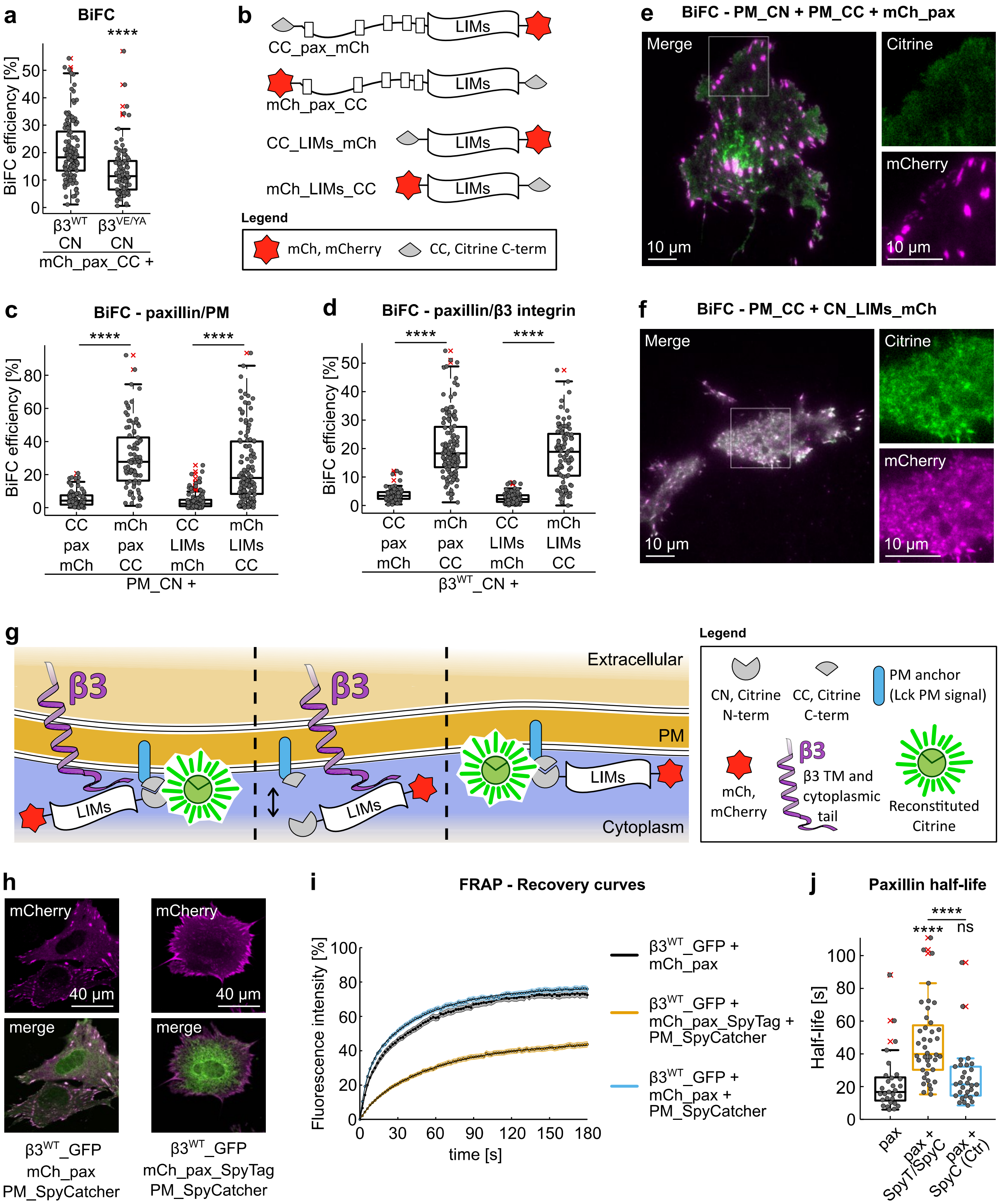

(**a**) Quantification of the BiFC efficiency upon co-expression of mCherry_paxillin_CC and $\beta 3^{WT}$_CN or $\beta 3^{VE/YA}$_CN in NIH-3T3 cells. Statistical analysis is provided in Supplementary Dataset 3. (**b**) Paxillin and LIMs fusion proteins tagged with mCherry and the citrine C-terminal fragment, tested in BiFC assays with alternatively PM_CN or $\beta 3^{WT}$_CN. (**c,d**) Quantification of the BiFC signal

generated by the co-expression of each of the paxillin constructs shown in figure **b** with (**c**) the plasma membrane-localized CN fragment, in Swiss-3T3 or with (**d**) the $\beta3^{WT}$ integrin C-terminally tagged with CN, in NIH-3T3 cells. Statistical analysis is provided in Supplementary Dataset 3. (**e**) Representative TIRF images of a triple positive Swiss-3T3 fibroblast, co-expressing mCherry_paxillin and the two complementary citrine fragments, each one fused to the PM-targeting peptide. While the BiFC signal is localized throughout to the plasma membrane, mCherry_paxillin only appears in the discreates spots of FAs. Brightness and contrast automatically optimized. (**f**) Example of a Swiss-3T3 fibroblast in which the co-expression of CN_LIMs_mCherry and PM_CC led to substantial BiFC and concomitant mis-localization of LIMs. Brightness and contrast automatically optimized. (**g**) Schematic representation of the possible scenarios, in terms of BiFC generation, upon co-expression of CN-tagged LIMs recombinant proteins and the PM-localized complementary citrine fragment. Left: the CN fragment at the C-terminus of LIMs leads to BiFC in adhesions. Middle: the CN fragment in front of LIMs is not compatible with BiFC in adhesions. Right: the CN fragment in front of LIMs can complement PM-localized CC outside adhesions. (**h**) Differential paxillin distribution among FAs, cytosol and PM, in the absence (left) and in the presence (right) of the irreversible fusion of the C-terminus to the PM_SpyCatcher. (**i**) Florescence Recovery After Photobleaching (FRAP) of paxillin localized to $\beta3^{WT}$_GFP-positive FAs, in the absence (mCherry_paxillin and mCherry_paxillin + PM_SpyCatcher, control) and in the presence (mCherry_paxillin_SpyTag + PM_SpyCatcher) of the irreversible fusion of the C-terminus to the PM_SpyCatcher. The mean value for each time point is represented with a dot, the standard error of the mean with a coloured background and the fitting of the double exponential function to the mean values with black a curve. (**j**) Box plot of the half-lives of paxillin in $\beta3^{WT}$_GFP-positive FAs. Statistical analysis is provided in Supplementary Dataset 4.

ns (not significant) $p > 0.05$; *, $p \leq 0.05$; **, $p < 0.01$; ***, $p < 0.001$; ****, $p < 0.0001$. Sample size, complete statistical analysis and *p* values are provided in tables in supplementary datasets.

Supplementary Fig. 5: Fitting of double decaying exponential curves to experimental protein dissociation rates.

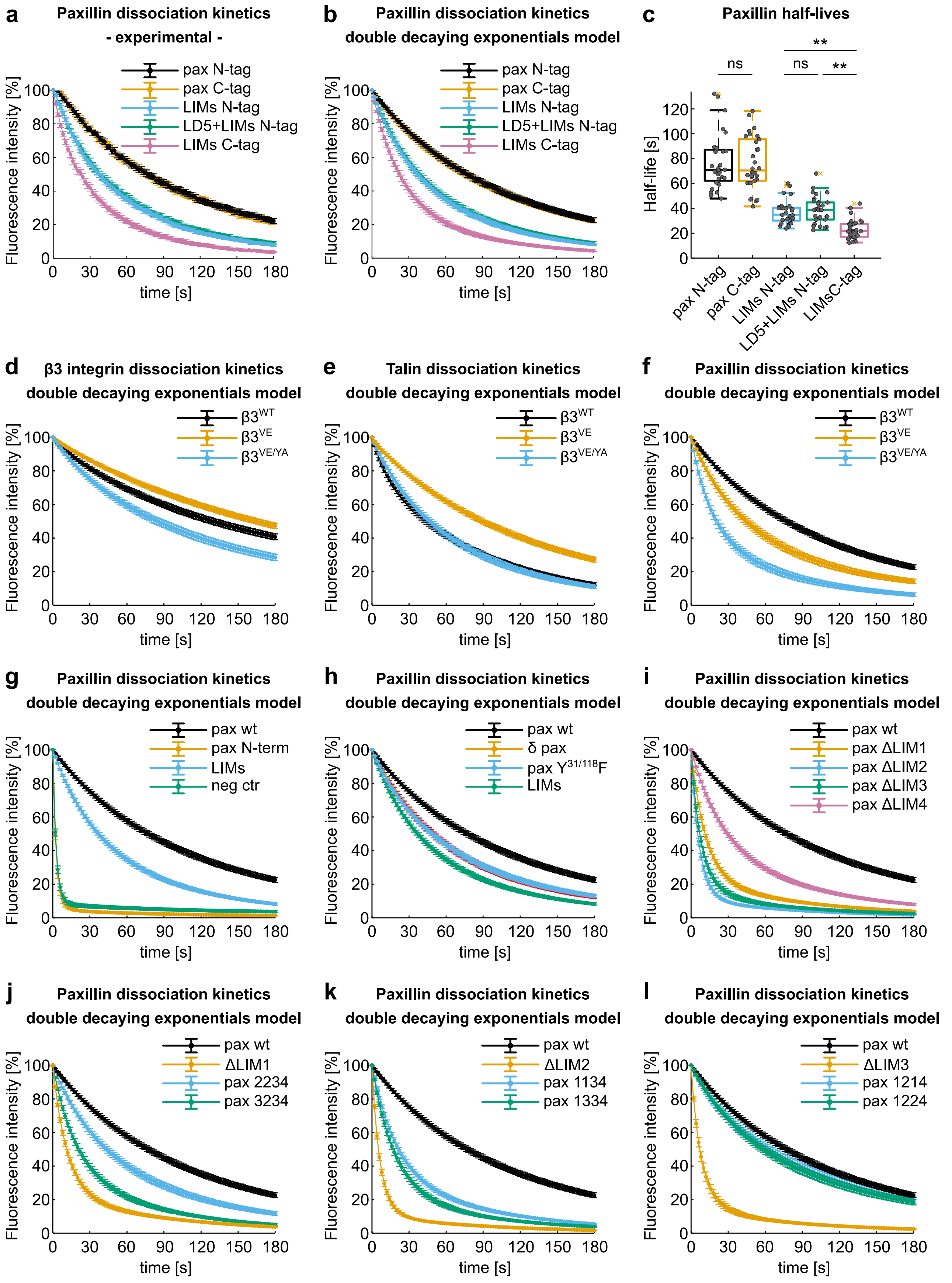

(**a**,**b**) Experimental and theoretical dissociation kinetics of PA-GFP_paxillin and LIM domains N- or C-terminally tagged from β3_mCherry-positive FAs. (**c**) Box plot of the half-lives of PA-GFP_paxillin and LIM domains N- or C-terminally tagged from β3_mCherry-positive FAs. Statistical analysis is provided in Supplementary Dataset 4. (**d**,**e**) Theoretical model of dissociation of (**d**) β3_PA-GFP integrins from mCherry_paxillin-positive FAs and of (**e**) PA-GFP_talin from β3_mCherry-positive FAs. (**f-l**) Theoretical model of dissociation of PA-GFP-tagged paxillin proteins from β3_mCherry-positive FAs.

ns (not significant) $p > 0.05$; *, $p \leq 0.05$; **, $p < 0.01$; ***, $p < 0.001$; ****, $p < 0.0001$. Sample size, complete statistical analysis and p values are provided in tables in supplementary datasets.

Supplementary Fig. 6: Photoactivation time series of paxillin in β3WT_mCherry-positive adhesions.

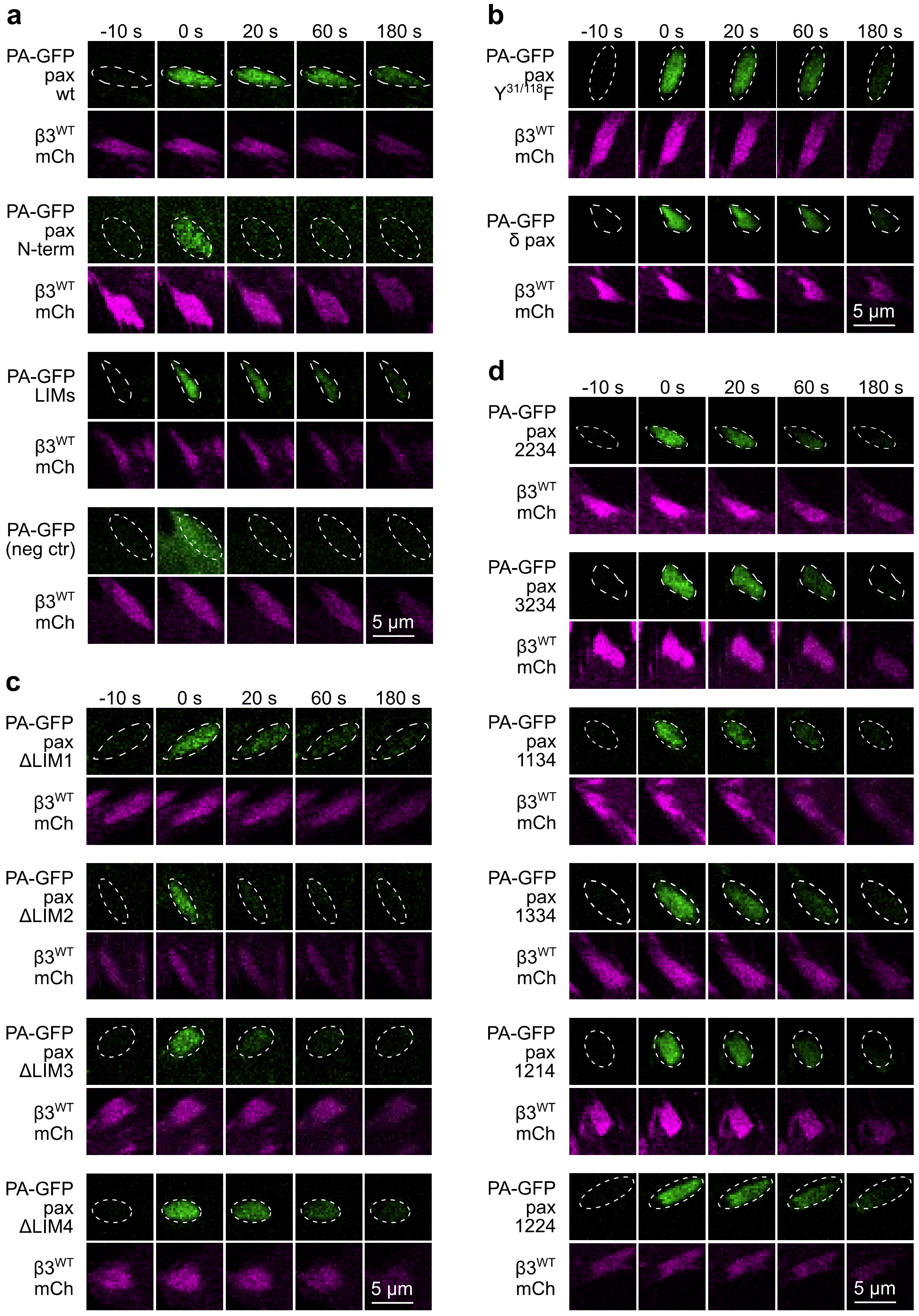

(**a**-**d**) Images of photoactivation time series of paxillin wt and mutants, transiently co-expressed with β3^{WT}_mCherry integrin in NIH-3T3 cells. Brightness and contrast of images in the green channel were adjusted to the image at t_0.

Supplementary Fig. 7: LIM deletion and replacement strategies.

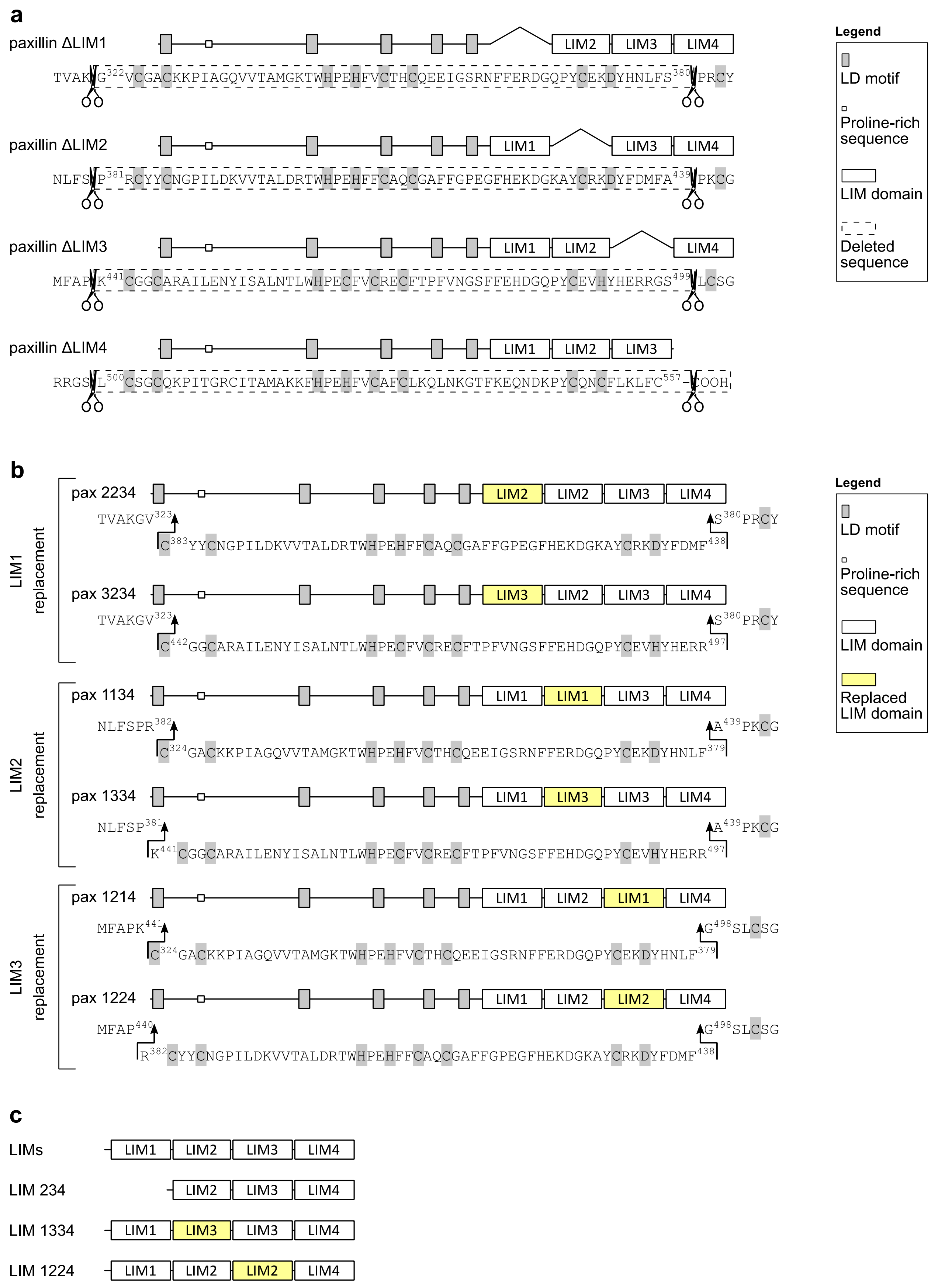

(**a**) Schematic representation of the paxillin deletion mutants, showing the exact amino acid sequence deleted. (**b**) Schematic representation of the paxillin mutants obtained by the LIM domain

replacement strategy. From the top line: replacements of LIM1, replacements of LIM2 and replacements of LIM3. The exact boundaries used for the replacement are shown in the sequences. (**c**) Schematic representation of the N-terminally GST-tagged LIM constructs used to perform biosensor experiments with His-tagged talin-head immobilized.

Supplementary Fig. 8: Recruitment of paxillin variants to β3 integrin-adhesions in different cell lines.

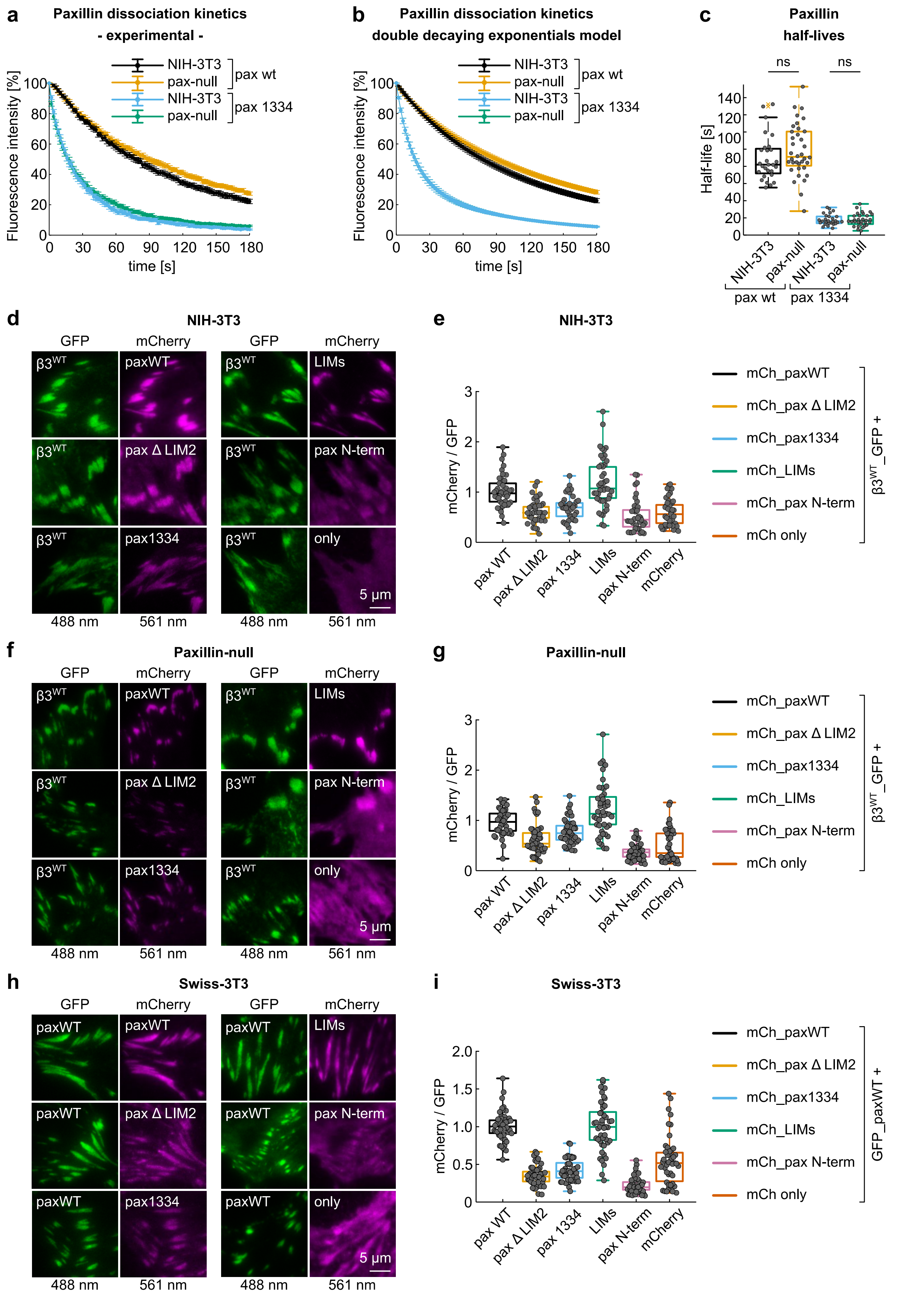

(**a**,**b**) Experimental and theoretical dissociation kinetics of PA-GFP_paxillin wt and 1334 mutant from β3_mCherry-positive FAs, in NIH-3T3 and paxillin-null fibroblasts. (**c**) Box plot of the half-lives of PA-GFP_paxillin wt and 1334 mutant from β3_mCherry-positive FAs, in NIH-3T3 and paxillin-null fibroblasts. Statistical analysis is provided in Supplementary Dataset 4. (**d**,**f**) Representative TIRF images of (**d**) NIH-3T3 and (**f**) paxillin-null fibroblasts co-expressing mCherry-tagged paxillin variants and $\beta3^{WT}$_GFP. (**e**,**g**) Quantification of the fluorescence ratio between mCherry_paxillin variants and $\beta3^{WT}$_GFP in GFP-positive FAs in (**e**) NIH-3T3 and (**g**) paxillin-null fibroblasts by TIRF microscopy. Descriptive statistics is provided in Supplementary Dataset 1. (**h**) Representative TIRF images of Swiss-3T3 fibroblasts co-expressing mCherry-tagged paxillin variants and GFP_paxillin wild-type. (**i**) Quantification of the fluorescence ratio between mCherry_paxillin variants and GFP_paxillin wild-type in GFP-positive FAs in Swiss-3T3 fibroblasts by TIRF microscopy. Descriptive statistics is provided in Supplementary Dataset 1.

ns (not significant) $p > 0.05$; *, $p \leq 0.05$; **, $p < 0.01$; ***, $p < 0.001$; ****, $p < 0.0001$. Sample size, complete statistical analysis and p values are provided in tables in supplementary datasets.
