# Supplementary material for: Structural and functional analysis of LIM domain-dependent recruitment of paxillin to αvβ3 integrin-positive focal adhesions
Source: Commun Biol. 2021 Mar 29;4:380. doi: 10.1038/s42003-021-01886-9 (PMC8007706; doi:10.1038/s42003-021-01886-9)
Supplement: Supplementary file 3 — Description Of Additional Supplementary Files [file 42003_2021_1886_MOESM3_ESM.pdf]

Description of Additional Supplementary Files

Supplementary Movies

Supplementary Movie 1 Live cell imaging of adhesions of NIH-3T3 co-expressing $\beta3^{WT}$_GFP and mCherry_paxillin.

Supplementary Movie 2 Live cell imaging of adhesions of NIH-3T3 co-expressing $\beta3^{VE}$_GFP and mCherry_paxillin.

Supplementary Movie 3 Live cell imaging of adhesions of NIH-3T3 co-expressing $\beta3^{VE/YA}$_GFP and mCherry_paxillin.

Supplementary Movie 4 Live cell imaging of adhesions of NIH-3T3 co-expressing $\beta3^{VE/YA}$_CN and mCherry_paxillin_CC. Green channel corresponds to the reconstituted citrine fluorophore.

Supplementary Datasets

Supplementary Dataset 1 Statistical analysis of protein fluorescence ratio in β3 integrin-adhesions.

Supplementary Dataset 2 Statistical analysis of the protein flow in adhesions.

Supplementary Dataset 3 Statistical analysis of BiFC experiments.

Supplementary Dataset 4 Statistical analysis of photoactivation and FRAP experiments.

Supplementary Dataset 5 Analysis of the proteomic data of Acyl-RAC experiments.

Supplementary Dataset 6 Source Data for graphs.
