# Supplementary material for: Structural and functional analysis of LIM domain-dependent recruitment of paxillin to αvβ3 integrin-positive focal adhesions
Source: Commun Biol. 2021 Mar 29;4:380. doi: 10.1038/s42003-021-01886-9 (PMC8007706; doi:10.1038/s42003-021-01886-9)
Supplement: Supplementary file 14 — Reporting Summary [file 42003_2021_1886_MOESM14_ESM.pdf]

nature research

Corresponding author(s): Bernhard Wehrle-Haller

Last updated by author(s): Feb 10, 2021

Reporting Summary

Nature Research wishes to improve the reproducibility of the work that we publish. This form provides structure for consistency and transparency in reporting. For further information on Nature Research policies, see our Editorial Policies and the Editorial Policy Checklist.

Statistics

For all statistical analyses, confirm that the following items are present in the figure legend, table legend, main text, or Methods section.

n/a	Confirmed	
☐	☒	The exact sample size (*n*) for each experimental group/condition, given as a discrete number and unit of measurement
☐	☒	A statement on whether measurements were taken from distinct samples or whether the same sample was measured repeatedly
☐	☒	The statistical test(s) used AND whether they are one- or two-sided<br>*Only common tests should be described solely by name; describe more complex techniques in the Methods section.*
☒	☐	A description of all covariates tested
☐	☒	A description of any assumptions or corrections, such as tests of normality and adjustment for multiple comparisons
☐	☒	A full description of the statistical parameters including central tendency (e.g. means) or other basic estimates (e.g. regression coefficient) AND variation (e.g. standard deviation) or associated estimates of uncertainty (e.g. confidence intervals)
☐	☒	For null hypothesis testing, the test statistic (e.g. *F*, *t*, *r*) with confidence intervals, effect sizes, degrees of freedom and *P* value noted<br>*Give P values as exact values whenever suitable.*
☒	☐	For Bayesian analysis, information on the choice of priors and Markov chain Monte Carlo settings
☒	☐	For hierarchical and complex designs, identification of the appropriate level for tests and full reporting of outcomes
☒	☐	Estimates of effect sizes (e.g. Cohen's *d*, Pearson's *r*), indicating how they were calculated

Our web collection on statistics for biologists contains articles on many of the points above.

Software and code

Policy information about availability of computer code

Data collection	Microscopy images were acquired with NIS Elements
Data analysis	Neurite function of Metamorph journal (MetaMorph 7.10).<br>Imaris 9.5 with custom Matlab script.<br>MATLAB 2019b.<br>Fiji: Phansalkar Auto Local Threshold (k = 0.1 , r = 0.1 and radius = 5) and Analyze Particle plugin.<br>Softwares used as stated in the material and methods.<br>Codes made accessible or available upon request to authors.

For manuscripts utilizing custom algorithms or software that are central to the research but not yet described in published literature, software must be made available to editors and reviewers. We strongly encourage code deposition in a community repository (e.g. GitHub). See the Nature Research guidelines for submitting code & software for further information.

Data

Policy information about availability of data

All manuscripts must include a data availability statement. This statement should provide the following information, where applicable:

- Accession codes, unique identifiers, or web links for publicly available datasets
- A list of figures that have associated raw data
- A description of any restrictions on data availability

Microscopy images that support the findings acquired during the current study are available in Yareta with the identifier doi: 10.26037/yareta:23jacb27ibdm5erlqxcdluzwrm. Raw proteomic data are available via ProteomeXchange with identifier PXD023810 (doi: 10.6019/PXD023810), while detailed sample handling by the Proteomics Core Facility is available in Yareta. List of figures that have associated raw data: 1b-n, 2a-b, 2d-h, 3a-i, 4a-h, 5b-e, 5g-i, 6c-g, 7b-l,

8b-h, 9b-e, 9g-j, Supplementary 1a-h, 2a-e, 3a-d, 4a, 4c-f, 4h-j, 5a-l, 6a-d, 8a-i. Source Data for graphs are available in Supplementary Data 6. Any other remaining information is available from corresponding author upon reasonable request.

Field-specific reporting

Please select the one below that is the best fit for your research. If you are not sure, read the appropriate sections before making your selection.

- [x] Life sciences
- [ ] Behavioural & social sciences
- [ ] Ecological, evolutionary & environmental sciences

For a reference copy of the document with all sections, see nature.com/documents/nr-reporting-summary-flat.pdf

Life sciences study design

All studies must disclose on these points even when the disclosure is negative.

Sample size	Sample size of each type of experiment was sufficient to accurately describe the data. Sample size is provided in Supplementary datasets as stated in figure captions.
Data exclusions	Outliers were excluded from statistical analyses, however they were represented in box and whisker plots.<br>For movies acquisition out of focus images were excluded if sudden and incompatible shifts were detected.
Replication	Whenever a measure is provided, it was obtained from at least 3 independent experiments
Randomization	Not applicable because no groups were created.
Blinding	Blind was not relevant because the same analysis has been applied to all the images acquired for a given type of experiment and not necessary for consistent or automatic analyses.

Reporting for specific materials, systems and methods

We require information from authors about some types of materials, experimental systems and methods used in many studies. Here, indicate whether each material, system or method listed is relevant to your study. If you are not sure if a list item applies to your research, read the appropriate section before selecting a response.

Materials & experimental systems

n/a	Involved in the study	
[ ]	[x]	Antibodies
[ ]	[x]	Eukaryotic cell lines
[x]	[ ]	Palaeontology and archaeology
[x]	[ ]	Animals and other organisms
[x]	[ ]	Human research participants
[x]	[ ]	Clinical data
[x]	[ ]	Dual use research of concern

Methods

n/a	Involved in the study	
[x]	[ ]	ChIP-seq
[x]	[ ]	Flow cytometry
[x]	[ ]	MRI-based neuroimaging

Antibodies

Antibodies used	Purified mouse monoclonal anti-paxillin (BD Biosciences 610051, 1:1000 dilution). Purified Rat Anti-Mouse CD29, clone 9EG7 (BD Pharmingen™, 1:600 dilution). Mouse monoclonal anti-vinculin (Sigma-Aldrich V9131, 1:600 dilution). Rabbit polyclonal anti-caveolin-1 (Santa Cruz Biotechnology sc-894, 1:4000 dilution). Rabbit polyclonal anti-mCherry (BioVision 5993-30T, 1:4000 dilution). Alexa Fluor 555 anti-mouse (ThermoFisher A-31570, 1:500 dilution). Alexa Fluor 633 anti-mouse (ThermoFisher A-21050, 1:500 dilution). Mouse monoclonal anti-GFP, clone B34 (BioLegend, 1:50˙000 dilution). HRP-conjugated anti-mouse (Jackson, 1:10˙000 dilution). HRP-conjugated anti-rabbit (Jackson, 1:10˙000 dilution).
Validation	Validated by the manufacturers. Antibodies widely used in our labs with no unspecific signals detected.

Eukaryotic cell lines

Policy information about cell lines

Cell line source(s)	Low β3 integrin-expressing NIH-3T3 cells and high β3 integrin-expressing Swiss-3T3 cells were validated previously (Pinon et al. 2014). Paxillin-null mouse fibroblasts provided by Prof. Daniel Bouvard (Centre de Recherche en Biologie cellulaire de Montpellier) and generated as described in the material and methods.
Authentication	Cell lines were not authenticated.

Mycoplasma contamination	We routinely visually inspected cells for mycoplasma contamination.
Commonly misidentified lines<br>(See ICLAC register)	N/A
